# Supplementary material for: Generation of Matrix Degradation Products Using an In Vitro MMP Cleavage Assay
Source: Int J Mol Sci. 2022 Jun 2;23(11):6245. doi: 10.3390/ijms23116245 (PMC9181598; doi:10.3390/ijms23116245)
Supplement: Supplementary file 1 [file ijms-23-06245-s001.zip › ijms-1638864-supplementary.pdf]

Supplemental Figures:

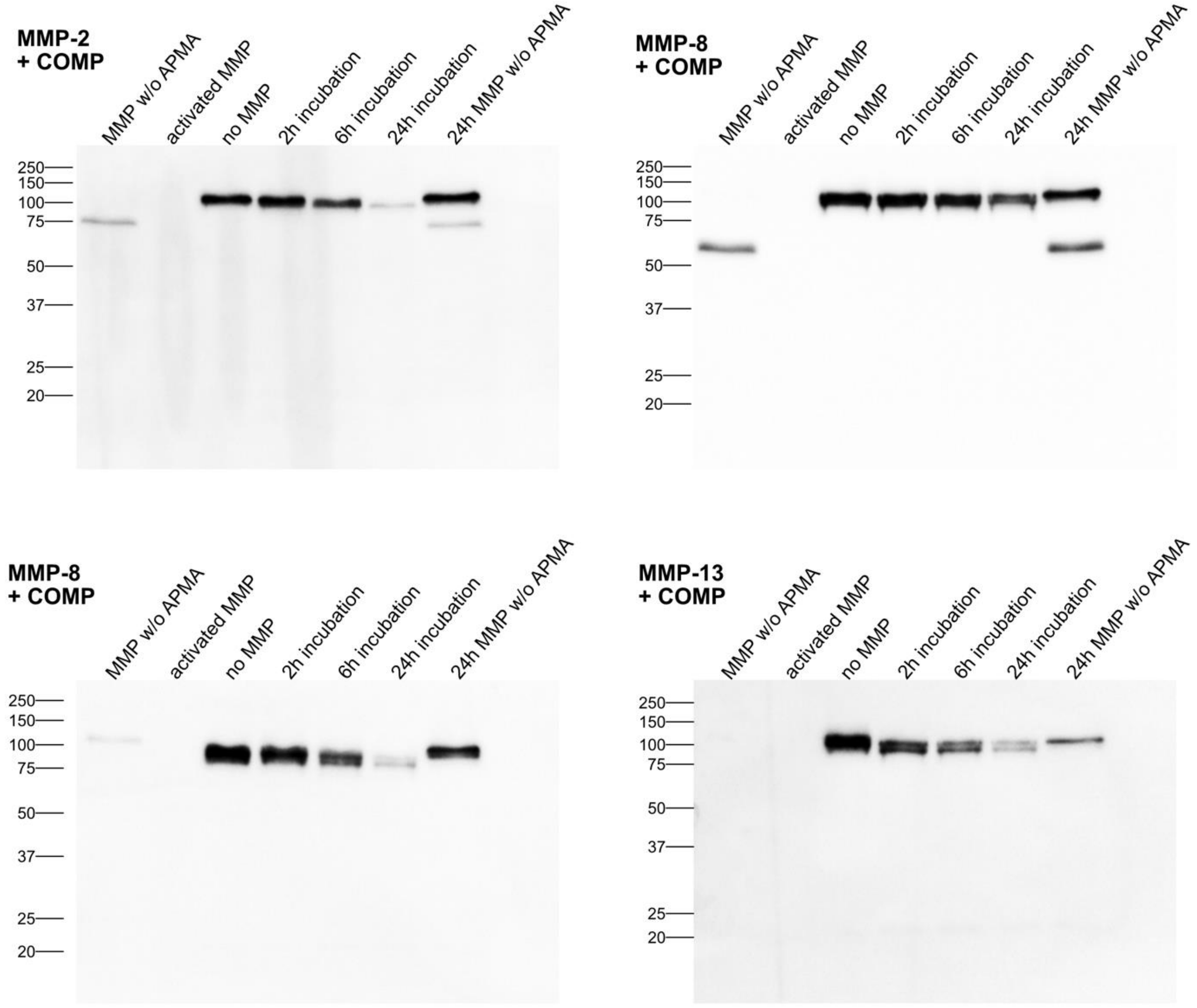

Figure S1: MMPs have to be activated by APMA for efficient proteolytic cleavage of COMP. COMP was incubated with activated or non-activated MMP for different time points. Efficient cleavage is indicated by a reduction of signal intensity and, in some cases, a shift towards a smaller size compared to the full-length protein without MMP (no MMP). Cleavage was observed with activated MMPs only.

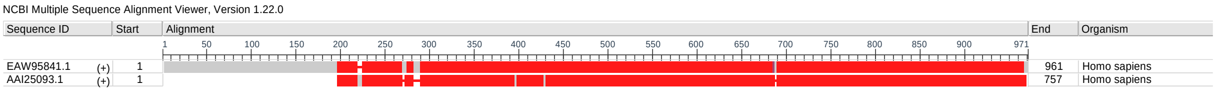


```
EAW95841.1  1    MLAPRGAAVLLLHLVLQRWLAAGAQATPQVFDLLPSSSQRLNPGALLPVLTDPALNDLYVISTFKLQTKSSATIFGLYSS  80
AAI25093.1       --------------------------------------------------------------------------------

EAW95841.1  81   TDNSKYFEFTVMGRLNKAILRYLKNDGKVHLVVFNNLQLADGRRHRILLRLSNLQRGAGSLELYLDCIQVDSVHNLPRAF  160
AAI25093.1       --------------------------------------------------------------------------------

EAW95841.1  161  AGPSQKPETIELRTFQRKPQDFLEELKLVVRGSLFQVASLQDCFLQQSEPLAATGTG------DFNRQFLGQMTQLNQLL  234
AAI25093.1  1    -----------------------------------MVPDTACVLLLTLAALGASGQGQSPLGSDLGPQMLRELQETNAAL  45

EAW95841.1  235  GEVKDLLRQQVKETSFLRNTIAECQACGPLKFQSPTPSTVVPPAPPAPPTRPPRRCDSNPCFRGVQCTDSRDGFQCGPCP  314
AAI25093.1  46   QDVRELLRQQVREITFLKNTVMECDACG---MQQSVRTGL-------PSVRPLLHCAPGFCFPGVACIQTESGARCGPCP  115

EAW95841.1  315  EGYTGNGITCIDVDECKYHPCYPGVHCINLSPGFRCDACPVGFTGPMVQGVGISFAKSNKQVCTDIDECRNGA--CVPNS  392
AAI25093.1  116  AGFTGNGSHCTDVNECNAHPCFPRVRCINTSPGFRCEACPPGYSGPTHQGVGLAFAKANKQVCTDINECETGQHNCVPNS  195

EAW95841.1  393  ICVNTLGSYRCGPCKPGYTGDQIRGC--KAERNCRNPELNPCSVNAQCIEERQGDVTCVCGVGWAGDGYICGKDVDIDSY  470
AAI25093.1  196  VCINTRGSFQCGPCQPGFVGDQASGCQRRAQRFCPDGSPSECHEHADCVLERDGSRSCVCAVGWAGNGILCGRDTDLDGF  275

EAW95841.1  471  PDEELPCSARNCKKDNCKYVPNSGQEDADRDGIGDACDEDADGDGILNEQDNCVLIHNVDQRNSDKDIFGDACDNCLSVL  550
AAI25093.1  276  PDEKLRCPERQCRKDNCVTVPNSGQEDVDRDGIGDACDPDADGDGVPNEKDNCPLVRNPDQRNTDEDKWGDACDNCRSQK  355

EAW95841.1  551  NNDQKDTDGDGRGDACDDDMDGDGIKNILDNCPKFPNRDQRDKDGDGVGDACDSCPDVSNPNQSDVDNDLVGDSCDTNQD  630
AAI25093.1  356  NDDQKDTDQDGRGDACDDDIDGDRIRNQADNCPRVPNSDQKDSDGDGIGDACDNCPQKSNPDQADVDHDFVGDACDSDQD  435

EAW95841.1  631  SDGDGHQDSTDNCPTVINSAQLDTDKDGIGDECDDDDDNDGIPDLVPPGPDNCRLVPNPAQEDSNSDGVGDICESDFDQD  710
AAI25093.1  436  QDGDGHQDSRDNCPTVPNSAQEDSDHDGQGDACDDDDDNDGVPD----SRDNCRLVPNPGQEDADRDGVGDVCQDDFDAD  511

EAW95841.1  711  QVIDRIDVCPENAEVTLTDFRAYQTVVLDPEGDAQIDPNWVVLNQGMEIVQTMNSDPGLAVGYTAFNGVDFEGTFHVNTQ  790
AAI25093.1  512  KVVDKIDVCPENAEVTLTDFRAFQTVVLDPEGDAQIDPNWVVLNQGREIVQTMNSDPGLAVGYTAFNGVDFEGTFHVNTV  591

EAW95841.1  791  TDDDYAGFIFGYQDSSSFYVVMWKQTEQTYWQATPFRAVAEPGIQLKAVKSKTGPGEHLRNSLWHTGDTSDQVRLLWKDS  870
AAI25093.1  592  TDDDYAGFIFGYQDSSSFYVVMWKQMEQTYWQANPFRAVAEPGIQLKAVKSSTGPGEQLRNALWHTGDTESQVRLLWKDP  671

EAW95841.1  871  RNVGWKDKVSYRWFLQHRPQVGYIRVRFYEGSELVADSGVTIDTTMRGGRLGVFCFSQENIIWSNLKYRCNDTIPEDFQE  950
AAI25093.1  672  RNVGWKDKKSYRWFLQHRPQVGYIRVRFYEGPELVADSNVVLDTTMRGGRLGVFCFSQENIIWANLRYRCNDTIPEDYET  751

EAW95841.1  951  FQTQNFDRFDN  961
AAI25093.1  752  HQLRQA-----  757
```

EAW95841.1 = Thrombospondin-4
AAI25093.1 = COMP

Figure S2: Sequence alignment of COMP and TSP-4. Red amino acids are conserved, blue indicates differences in sequence.
